# Photocatalytic Activity and Biocide Properties of Ag–TiO_2_ Composites on Cotton Fabrics

**DOI:** 10.3390/ma16134513

**Published:** 2023-06-21

**Authors:** Uriel Chacon-Argaez, Luis Cedeño-Caero, Ruben D. Cadena-Nava, Kendra Ramirez-Acosta, Sergio Fuentes Moyado, Perla Sánchez-López, Gabriel Alonso Núñez

**Affiliations:** 1 Departamento de Ingeniería Química, Facultad de Química, Universidad Nacional Autónoma de México, Mexico City 04510, Mexico; uurielca@gmail.com; 2 Departamento de Bionanotecnología, Centro de Nanociencias y Nanotecnología, Universidad Nacional Autónoma de México, Ensenada 22800, Mexico; rcadena@ens.cnyn.unam.mx (R.D.C.-N.); g3_rama14@ens.cnyn.unam.mx (K.R.-A.); 3 Centro de Investigación Científica y de Educación Superior de Ensenada, Ensenada 22860, Mexico; 4 Departamento de Nanocatálisis, Centro de Nanociencias y Nanotecnología, Universidad Nacional Autónoma de México, Ensenada 22800, Mexico; perlasanchez@ens.cnyn.unam.mx (P.S.-L.); galonso@ens.cnyn.unam.mx (G.A.N.)

**Keywords:** Ag nanoparticles, Ag–TiO_2_ nanoparticles, cotton fabrics, photocatalytic activity, biocide activity, *E. coli*, *S. aureus*

## Abstract

Composites of Ag and TiO_2_ nanoparticles were synthesized in situ on cotton fabrics using sonochemical and solvothermal methods achieving the successive formation of Ag-NPs and Ti-NPs directly on the fabric. The impregnated fabrics were characterized using ATR-FTIR spectroscopy; high-resolution microscopy (HREM); scanning electron microscopy coupled with energy-dispersive X-ray spectroscopy (SEM-EDS); Raman, photoluminescence, UV-Vis, and DRS spectroscopies; and by tensile tension tests. Results showed the successful formation and impregnation of NPs on the cotton fabric, with negligible leaching of NPs after several washing cycles. The photocatalytic activity of supported NPs was assessed by the degradation of methyl blue dye (MB) under solar and UV irradiation revealing improved photocatalytic activity of the Ag–TiO_2_/cotton composites due to a synergy of both Ag and TiO_2_ nanoparticles. This behavior is attributed to a diminished electron–hole recombination effect in the Ag–TiO_2_/cotton samples. The biocide activity of these composites on the growth inhibition of *Staphylococcus aureus* (Gram+) and *Escherichia coli* (Gram−) was confirmed, revealing interesting possibilities for the utilization of the functionalized cotton fabric as protective cloth for medical applications.

## 1. Introduction

The development of nanotechnology has played a crucial role in the design, synthesis, and application of different types of nanomaterials [1]. Nowadays, semiconductor and metal nanoparticles (NPs) are widely used for healthcare, agriculture, ecology, energy, and catalysis due to their unique optoelectronic, physicochemical, antimicrobial, and catalytic properties [2,3,4]. Titania (TiO_2_) is the semiconductor most used in photocatalysis due to its low cost of production, good availability, high stability, and high activity. It is a type n semiconductor, with some ionic character where the 3d orbitals of Ti^4+^ contribute to the conduction band, while the p orbitals of O^2−^ contribute to the valence band [5]. However, titania is susceptible to present the phenomenon of electron/hole (e^−^/h^+^) recombination, diminishing its photocatalytic properties. Nevertheless, the addition of metallic nanoparticles can contribute to decreasing the e^−^/h^+^ recombination because they act as electron traps, and they can even adsorb photons with less energy than titania [6]. Therefore, the combined use of semiconductors and metallic nanoparticles can provide improved photocatalytic properties.

The incorporation of nanoparticles of TiO_2_ (TiO_2_-NPs) on fabrics induces antiviral properties as well as UV protection and hydrophobicity/hydrophilicity, which can act as flame retardants and provide self-cleaning properties [7,8]. These antibacterial properties are related to reactive oxygen species (ROS) formed in the presence of UV irradiation, although antibacterial activity has also been observed under dark conditions and the active species have not been elucidated [9].

On the other hand, it is well known that silver has antibacterial properties per se [10,11]. For many centuries, silver has been used as an antimicrobial agent to prevent the spread of infections. There is a lot of literature devoted to the antibacterial applications of silver nanoparticles [10,12,13,14,15]. The application of Ag nanoparticles to face masks by chemical reduction has been reported in [16] and [17]. These masks were used for the inhibition of bacteria Gram + (*Staphylococcus aureus*) and Gram − (*Escherichia coli*), revealing a positive effect of NPs on both types of bacteria.

Regarding the synthesis of TiO_2_-NPs, Guo et al. [18] reported a low-temperature method to produce nanocrystalline TiO_2_ by means of sonochemical treatment during the hydrolysis of titanium alkoxides. Prasad et al. [19] also prepared TiO_2_-NPs at low temperatures by using a sol–gel method under ultrasound. Other types of nanoparticles have been incorporated into cotton fabrics by using ultrasonic irradiation, such as Ag nanoparticles [20], ZnO nanoparticles [21], CuO nanoparticles [22], and TiO_2_-NPs [23,24]. Additionally, the immobilization of TiO_2_-NPs on cotton, obtaining the anatase phase without requiring subsequent heating, and using the ultrasound-assisted sol–gel method was reported. This method showed high resistance of the fabrics to leaching under several wash cycles, thus maintaining their self-cleaning properties and UV protection [24,25]. The incorporation of nanoparticles in textiles is of great importance for the development of personal protection equipment (PPE) to face pandemics such as COVID-19 in a more efficient way [26].

The objective of this work was to reveal the effect of nanocomposites formed by TiO_2_ with silver nanoparticles directly deposited in Indiolino fabrics with respect to the photocatalytic degradation of methyl blue and the inhibition growth of Gram + or Gram − bacteria, in the presence of UV and visible light. The optimization of these nanocomposites directly deposited on fabrics can contribute to developing better protective clothes for personal or medical use.

This research reports the in situ immobilization of NPs of Ag and TiO_2_ on cotton fabric obtained using ultrasound-assisted and solvothermal successive methods. The appropriate formation and impregnation of the Ag and TiO_2_ nanoparticles on the surface of cotton fabrics were analyzed using ATR-FTIR, Raman, HREM, and SEM-EDS. Additionally, the photocatalytic activity of the NPs impregnated on cotton fabrics using aqueous solutions of methylene blue (MB) dye under solar and UV irradiation was tested. Finally, the biocide activity of the Ag–TiO_2_/cotton fabric composite textiles was determined based on the growth inhibition of *Staphylococcus aureus* and *Escherichia coli* in liquid culture and LB agar plates.

## 2. Materials and Methods

### 2.1. Materials

Commercial Indiolino fabric (100% cotton, 175 g/m^2^) was used as a model textile. All precursor reagents were provided by Merck and used without further purification: titanium butoxide (97%), terbutanol (99%), acetic acid (99.7%), AgNO_3_ (99%), NaBH_4_ (99%), and methylene blue (95%). Squares (5 × 5 cm) of Indioline were used for impregnation. Prior to impregnation, the Indioline textile was washed with a nonionic detergent and dried at 70 °C for 24 h.

### 2.2. Synthesis and Incorporation of Ag and TiO_2_ NPs on Cotton Fabrics

**Scheme 1:**
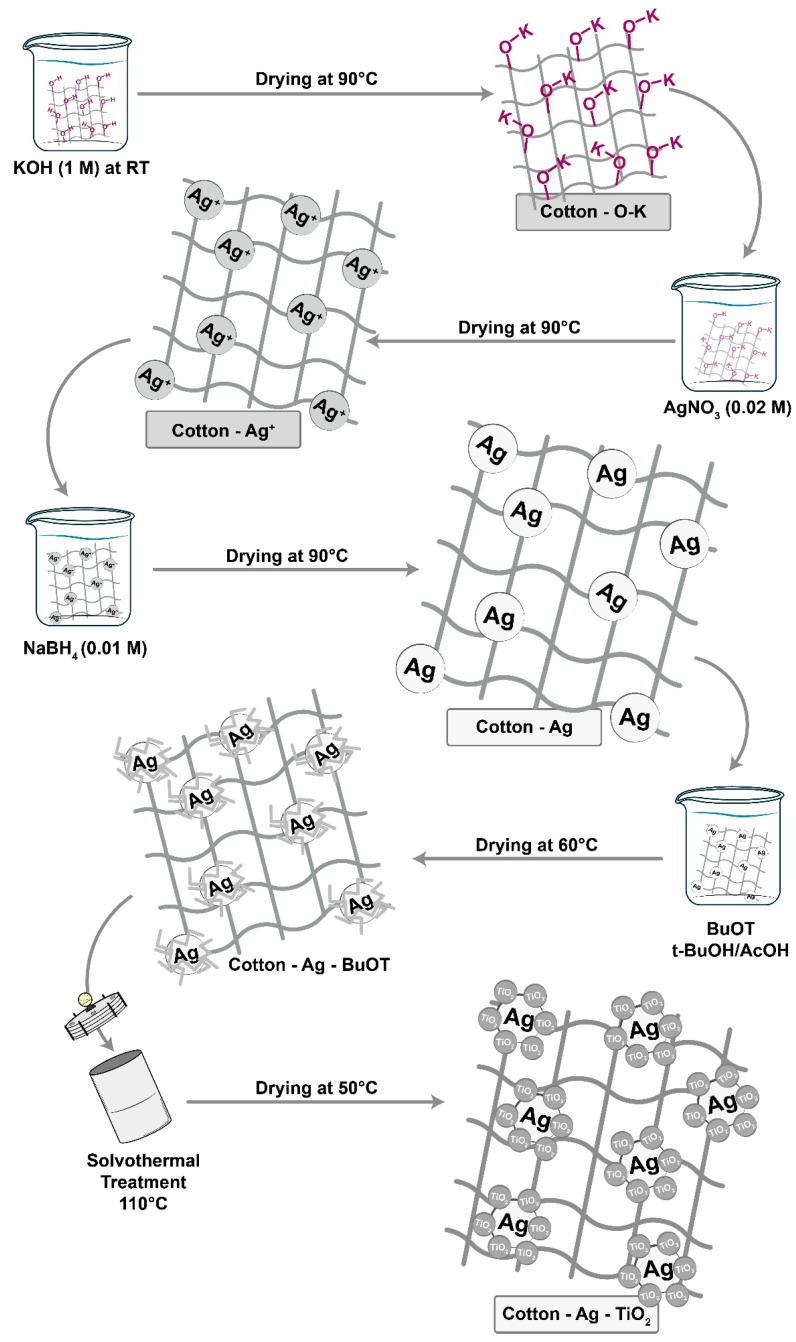
Illustration of the different steps involved in the preparation of Ag–TiO_2_ NPs on cotton.

The impregnation procedure was optimized by varying the loading of Ag and TiO_2_ NPs as well as the temperature, time, and washing. Prior to impregnation, the textile was functionalized to assure good anchoring of the nanoparticles (NPs), according to the method described by Dong and Hinestroza [27]. This method consists of treating the fabric with a KOH solution (1 M), at room temperature under constant stirring for 10 min; then, the fabric was rinsed with distilled water and dried at 90 °C for 45 min. Finally, the preparation of the Ag–TiO_2_ on cotton samples was carried out according to Scheme 1.

#### 2.2.1. Synthesis of Ag NPs on Cotton Fabrics

As Scheme 1 indicates, the first step was the generation of Ag NPs by immersion of the cotton fabric (5 cm × 5 cm) into 100 mL of AgNO_3_ solution in water (0.02 M) for 30 min. Then, the fabric was dried at 90 °C for 45 min and immersed in a solution of NaBH_4_ (0.01 M) for 30 min to reduce the adsorbed Ag^+^ ions. Later, the sample was copiously rinsed with water and then dried at 90 °C for 45 min.

#### 2.2.2. Synthesis of TiO_2_ NPs on Cotton Fabrics

NPs of TiO_2_ on cotton fabrics (Ti-NPs) were obtained by solvothermal synthesis [24], which is briefly described below. Indioline squares (5 × 5 cm) were dried at 70 °C for 4 h to desorb water. Then, they were dipped in a solution of titanium butoxide (BuOT, 1 wt.%) in a Mixture of terbutanol and acetic acid (90/10 wt.%) for 12 h at room temperature. Samples were subsequently transferred into an oven and dried at 60 °C for 2 h. They were then treated in a Teflon-lined stainless-steel autoclave, which was half-filled with water, at 110 °C for 3 h. The samples were finally dried at 50 °C for 2 h.

#### 2.2.3. Synthesis and Incorporation of Ag and TiO_2_ NPs on Cotton Fabrics

Composites of Ag and TiO_2_ were prepared by successive impregnation of Ag-NPs on cotton and subsequent impregnation of TiO_2_ NPs using the methods described (see Scheme 1).

### 2.3. Photocatalytic Tests

#### 2.3.1. Methylene Blue Degradation by UV Light

The test used to evaluate the photocatalytic activity of the Ag–TiO_2_/cotton samples was the MB degradation reaction under UV light. The MB reagent was impregnated on the cotton fabrics (3 mL, 5 ppm) before being irradiated with the UV light (mercury lamp) source (254–320 nm) with an irradiance of 10 mW/cm^2^. A UV-Vis DRS spectrophotometer was used to measure the maximum absorbance value (C_0_, t = 0) and the absorption spectra (C_t_) as a function of the time of UV light exposure (15, 30, 60, and 120 min). The MB degradation rate was obtained from the relative C/C_0_ values, where C represents the MB concentration at time t [28].

#### 2.3.2. Methylene Blue Degradation Tests Using Solar Irradiation

The photocatalytic properties of cotton fabrics functionalized with NPs of Ag and TiO_2_ were also assessed for MB degradation using solar irradiation. Briefly, the experimental procedure was as follows: 2.5 cm × 2.5 cm pieces of cotton Indioline samples were impregnated with an MB solution (3 mL, 5 ppm). Then, the cotton samples were placed in a container that was exposed to sunlight.

The average natural daylight irradiance was 3 mW/cm^2^, in winter from 9 am to 3 pm in Mexico City. The initial concentration of MB (C_0_) on the cotton surface at 15, 30, 60, and 120 min of exposition to sunlight was assessed using UV–vis DRS spectroscopy. MB degradation was determined by the relation C/C_0_ as a function of time, where C_0_ and C represent the initial MB concentration on the impregnated cotton pieces and the MB concentration at time t, respectively.

### 2.4. Washing Tests

The impregnated fabrics were subjected to washing cycles in agreement with the standard FZ/T 73023-2006 for textiles. The samples were placed in an ultrasound device of 100 Hz at 40 °C for 5 min; then, they were rinsed with distilled water. Finally, the cotton samples and the washing water were both analyzed using UV-Vis and UV-Vis DRS spectroscopy to determine the leaching of Ag and TiO_2_ NPs [25].

### 2.5. Characterization

The impregnated samples were characterized using attenuated total reflectance infrared spectroscopy (ATR-FTIR), carried out in a Nicolet 6700 FT-IR spectrometer (Thermo Fisher Scientific Inc., Waltham, MA, USA), to determine the incorporation of NPs in the fabrics. The samples were also characterized using Raman spectroscopy and DxR Raman microscopy (exciting laser line of 785 nm) in a Thermo Scientific instrument Horiba, Lyon, France to evaluate the characteristic vibrations of the NPs. The synthesis solutions and the water from the washing tests were analyzed using UV-Vis spectroscopy, at RT (Varian Cary 5, Agilent Technologies Inc., Santa Clara, CA, USA), from 200 to 1100 nm, to assess the impregnated and leached amount of NPs, respectively. The morphology and composition of the impregnated fabrics were characterized using scanning electron microscopy (SEM) and energy dispersive spectroscopy (EDS) with a Jeol JSM-5900 LV microscope (Jeol USA Inc., Peabody, MA, USA). The surface area of cotton samples was determined by N_2_ adsorption–desorption isotherms at −196 °C on a Tristar Micrometrics apparatus (Micromeritics Instruments Corp., Norcross, GA, USA); before the nitrogen adsorption, the samples were degassed for 8 h under vacuum at 350 °C. The mechanical resistance of all fabrics, before and after Ag and TiO_2_ NPs impregnation, was evaluated on a digital traction force gauge, LZKW. The MB degradation on the surface of the cotton pieces was analyzed using UV-Vis DRS (Perkin Elmer Lambda 365, Perkin Elmer Inc., Waltham, MA, USA). The photoluminescence spectra of the fabrics were determined at room temperature using a spectrofluorometer model JASCO FP-8550 (Jasco Inc., Easton, MD, USA) in conjunction with an ISF-134 integration sphere.

### 2.6. Biocide Activity

The bacterial strains used in this work were *Escherichia coli* DH5α Thermo Fisher EC0112 and *Staphylococcus aureus* ATCC 25923. The LB broth and LB agar were prepared following Miller’s LB protocol (10 g/L NaCl, 5 g/L yeast extract, 10 g/L tryptone for liquid media, and 15 g/L agar were also added to prepare solid media). Reagents used to prepare both media were purchased from Merck (Sigma-Aldrich, St. Louis, MO, USA).

Biocide activity experiments were conducted by cutting 0.5 cm × 0.5 cm squares from the three samples: regular cotton fabric, Ag-NPs, and AgTi-NPs composites (impregnated fabrics). The samples were then inoculated by immersion in 106 CFU/mL *E. coli* or *S. aureus* cultures. A kanamycin control was prepared by immersing the Indiolino cotton fabric in a 1 mg/mL kanamycin solution, followed by inoculation with the desired bacterial culture. The inoculated fabrics were then deposited on top of a glass microscope slide (Fisher Brand) and covered with a second slide. This setup allowed for the exposure of the fabrics to varying periods of sunlight. Group A was not exposed to sunlight (t = 0 min), Group B was exposed to 20 min of sunlight on each side (t = 20 min), and Group C was exposed to 60 min of sunlight on each side (t = 60 min). The sunlight and UV AB radiation were measured using a URCERI light meter and a UV AB detector. After exposure to sunlight, the fabrics were subdivided into two groups. The first group was deposited into a 96-well plate where each fabric sample was submerged in a well with 200 µL of LB medium. The well plate was then incubated at 37 °C with constant agitation at 250 rpm for 18 h. Afterward, the fabrics were removed from the wells and bacterial growth was determined by measuring optical density at 600 nm on a UV-Vis spectrometer. The second group of fabrics were incubated on LB agar plates at 37 °C. Photographs of the plates were taken at 20 and 40 h of incubation to evaluate bacterial growth around the fabrics at different time intervals. All experimental conditions were performed in triplicate.

## 3. Results and Discussion

The present research studied the photocatalytic and biocide properties of Ag–TiO_2_ composites on cotton fabrics. These composites were characterized by means of mechanical, structural, physicochemical, and spectroscopic techniques.

### 3.1. Tensile Properties of Fabrics before and after the Preparation Tests

The tensile properties of fabrics such as thread diameter, grammage, and tensile strength were measured for bare and impregnated Indiolino. In a previous work [24], Indiolino showed the following characteristics: 0.4 m^2^/g of surface area, 0.38 mm of lattice thread diameter, grammage of 175 g/m^2^, and 9.90 N of tensile strength.

Indiolino was immersed successively in an ultrasound bath with KOH, AgNO_3_, NaBH_4_, and BuOT solutions under different conditions, with intermediary drying steps; finally, it was treated in a solvothermal process at 110 °C. After the impregnation procedure, the mechanical properties were measured and the tensile strength of the fabrics was only 5% less resistant than the original one. These results indicated that in situ preparation of NPs on Indiolino did not cause significant damage to the structure of the cotton.

### 3.2. TEM, SEM-EDS, and Elemental Mapping

**Figure 1:**
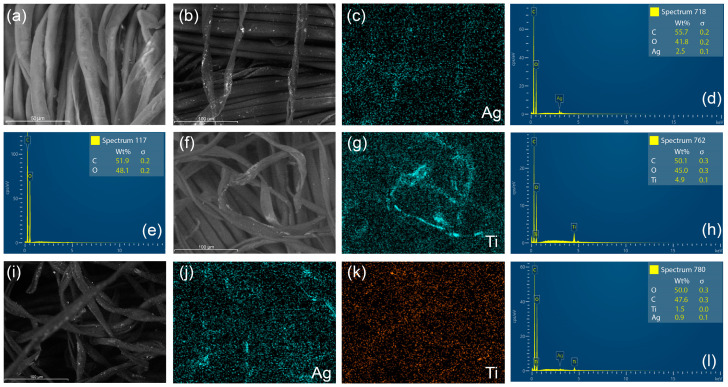
SEM-EDS micrographs: (**a**) Cotton-alone fabric; (**b**) Ag-NPs samples; (**f**) Ti-NPs samples; (**i**) Ag-Ti-NPs samples. Elemental mapping: (**c**) Ag in the Ag-NPs sample; (**g**) Ti in the Ti-NPs sample; (**j**) Ag in the AgTi-NPs sample; (**k**) Ti in the AgTi-NPs sample. EDS spectrum: (**d**) Ag-NPs sample; (**e**) Cotton-alone fabrics; (**h**) Ti-NPs sample; (**l**) AgTi-NPs sample.

Figure 1 shows the SEM images of the following samples: cotton-alone fabric (Figure 1a), Ag-NPs on cotton (Figure 1b), Ti-NPs on cotton (Figure 1f), and AgTi-NPs on cotton (Figure 1i). All SEM images of the samples with NPs show that the structures of the cotton microfibers remain intact despite the different chemical and thermal treatments they underwent to incorporate the NPs.

Figure 1b shows nanoparticles on cotton threads, suggesting the correct synthesis of the Ag-NPs on the fabrics. In addition, according to the elemental mapping (Figure 1c), the distribution of these particles on the surface of the fabrics was adequate. The same behavior was observed for the Ti-NPs sample, as shown in the SEM micrograph (Figure 1f) and the elemental mapping in Figure 1g. From the EDS elemental analysis, the average loadings of Ag and Ti were 2.5 and 4.9 wt.% for the Ag-NPs and Ti-NPs samples, respectively, meanwhile for the AgTi-NPs sample, the elemental loading decreased to 0.9 wt.% for Ag and 1.5 wt.% for Ti. This difference can be attributed to the fact that some NPs weakly anchored to the cellulose fiber may be removed in an additional step of the nanocomposite synthesis. Additionally, the elemental mapping (Figure 1j,k) of the AgTi-NPs sample shows the adequate distribution of Ag and Ti species.

**Figure 2:**
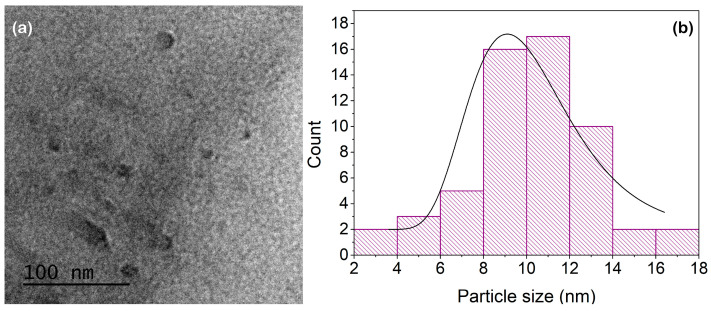
(**a**) TEM micrograph of Ti-NPs sample; (**b**) Lognormal particle size distribution curves of Ti-NPs.

**Figure 3:**
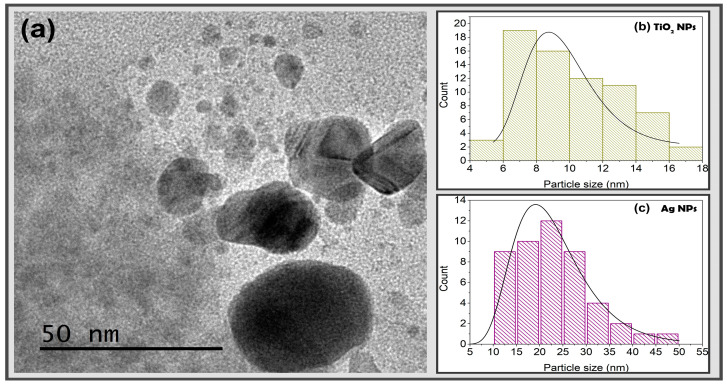
(**a**) TEM micrograph of AgTi-NPs sample and lognormal particle size distribution curves of Ti-NPs (**b**) and Ag-NPs (**c**).

The morphology and size of the nanostructures were examined using transmission electron microscopy (TEM). The results are shown in Figure 2 and Figure 3 for Ti-NPs and AgTi-NPs samples, respectively. The darker and larger particles were assigned to Ag-NPs, while the smaller ones with a lighter shade correspond to TiO_2_-NPs. In both samples, an almost spherical morphology was observed. Furthermore, in the AgTi-NPs sample micrograph (Figure 3a), the Ti-NPs show agglomerations in some areas of the cotton fibers. The sizes of the Ag and TiO_2_ nanoparticles were calculated using the ImageJ software to construct the particle size distribution curves (lognormal distribution). The results are shown in Figure 2b and Figure 3b,c. The average size of the Ag-NPs was 21.6 ± 1.4 nm for Ag-NPs and AgTi-NPs samples, whereas for the TiO_2_-NPs it was 10.3 ± 2.76 nm in the sample without Ag and 9.6 ± 1.3 nm for the Ag–TiO_2_ composite. The interface formed between these nanoparticles suggests the presence of an energy level (attributed to the Ag-NPs) below the conduction band of the TiO_2_-NPs, resulting in a reduction in the value of the forbidden gap.

### 3.3. Attenuated Total Reflectance Infrared Spectroscopy (FTIR-ATR)

**Figure 4:**
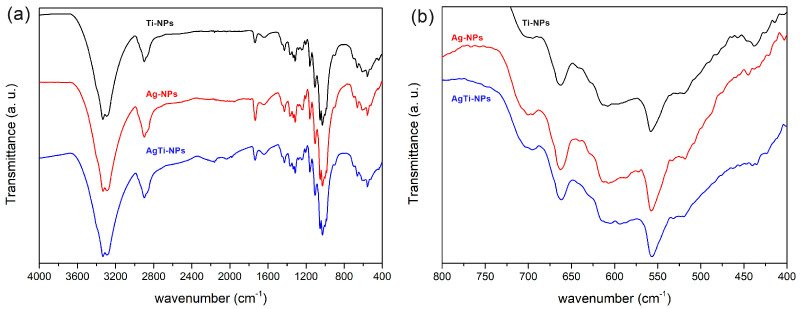
(**a**) FTIR-ATR spectra of the Ti-NPs, Ag-NPs, and AgTi-NPs samples. (**b**) Closer view of (**a**) in the range of 800 to 400 cm^−1^.

The modified cotton fabrics were analyzed using infrared spectroscopy (FT-IR ATR). In Figure 4, the bands near 3333 and 1334 cm^−1^ are assigned to hydroxyl groups [29,30]. Meanwhile, the bands at 2898, 1427, and 1314 cm^−1^ are attributed to the different types of vibrations of the CH_2_ bond. The bands at 1736 and 1369 cm^−1^ correspond to the carbonyl and CH bonds. The bands between the 1204 to 1000 cm^−1^ region correspond to C–O bonds while the band at 900 cm^−1^ corresponds to β-cellulose bonds. Figure 4b shows the region between 800 and 400 cm^−1^ where, according to Lu et al. (2018), the vibrational bands of the Ti–O–Ti bonds are observed. Other bands identified in this region were at 694 cm^−1^ [31] corresponding to CH_2_ rocking in crystalline cellulose Iβ and the bands at 662 and 558 cm^−1^ attributed to C–OH bending [32,33].

Furthermore, the Ti-NPs showed a band at 437 cm^−1^ which is attributed to the anatase crystalline phase of TiO_2_ [34,35]. This band is found to be less intense in the AgTi-NPs sample, which can be attributed to the fact that the previous monolayer of Ag-NPs on the surface of the fabric affects and limits the amount of TiO_2_ particles that can be deposited on the surface. According to SEM-EDS analysis, this effect is attributed to the decrease in Ti loading.

### 3.4. X-ray Diffraction (XRD) and Raman Spectroscopy

**Figure 5:**
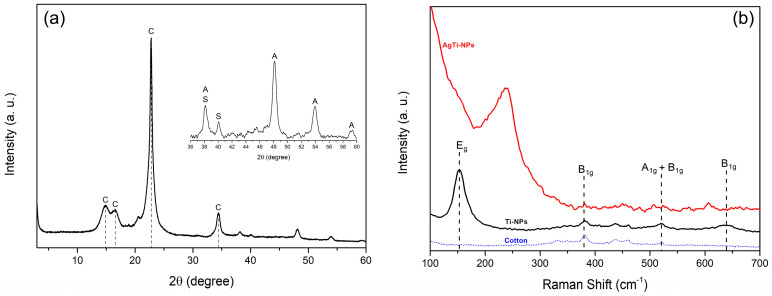
(**a**) XDR pattern of AgTi-NPs sample, where C = cellulose, S = silver, and A = anatase. (**b**) Raman spectra of AgTi-NPs, Ti-NPs, and cotton fabric.

Figure 5 shows the XRD pattern of the AgTi-NPs sample, where the main peaks at 15.14°, 16.25°, 22.75°, and 34.39° are attributed to the IB cellulose fibers forming a crystalline microstructure [36].

The weak diffraction at 40.16° corresponds to metallic silver particles with hexagonal structure (ICD No. 064707) [37], and the crystallite size calculated using the Debye–Scherrer equation gave a value of 34.2 nm. The interface formed between the Ag-NPs and TiO_2_ on the surface of the cotton fibers modified the characteristic diffraction pattern of TiO_2_ [38]. Only peaks at 48.15°, 53.96°, and 59.27° were identified, corresponding to the anatase crystalline phase of TiO_2_ (PDF 03-065-5714) with a calculated crystallite average size of 15 nm. The weak sharp peak at 38.2° was considered to be a contribution between the Ag-NPs (ICDS No. 652871) [39] and the anatase phase of TiO_2_. For Ti-NPs and Ag-NPs samples, similar XRD patterns were obtained where only anatase and silver phases were observed, respectively.

The Raman spectrum of the Ti-NPs sample (Figure 5b) showed anatase phase peaks at 153 (E_g_), 380 (B_1g_), 520 (A_1g_ + B_1g_), and 639 cm^−1^ (B_1g_). Meanwhile, the small peaks between 420 and 500 cm^−1^ corresponded to cellulose. The AgTi-NPs sample showed a broad complex band in the range of 220–300 cm^−1^ due to multiphoton scattering processes [40]. Since all the vibrations in the Raman spectrum move mainly oxygen atoms, the introduction of silver atoms changed the local coordination of oxygen around Ti^4+^. To maintain charge neutrality by silver doping, oxygen vacancies are created in the TiO_2_ lattice. When the silver ion replaces the Ti^4+^ ion during doping, the Ti–O–Ti bonds are distorted, and new Ag–O–Ti or Ag–O–Ag complex bonds are formed [40]. This complex band confirmed the adequate synthesis of Ag–TiO_2_ nanocomposite on cotton fabric.

### 3.5. UV-Vis Studies: Spectra of Reaction Solutions and Diffuse Reflection Spectra (DRS) of Fabrics

**Figure 6:**
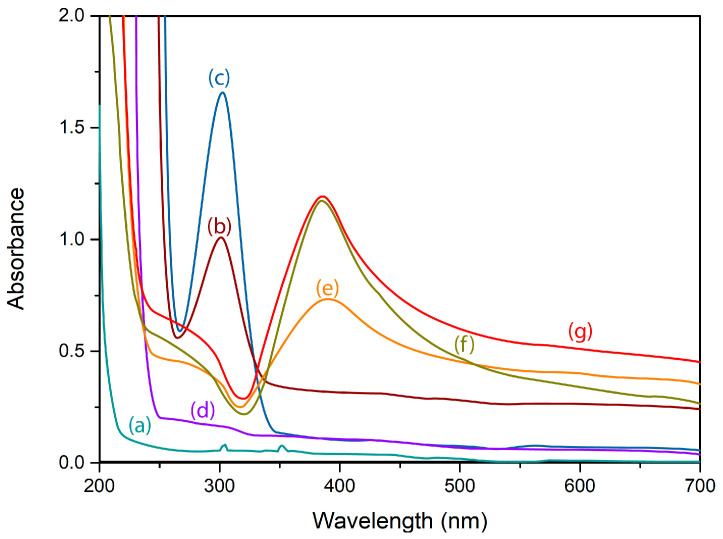
UV-Vis spectra of the NaBH_4_ and AgNO_3_ solutions: (**a**) [NaBH_4_] = 0.02 M; (**b**) [AgNO_3_] = 0.01 M; (**c**) [AgNO_3_] = 0.025 M. Solutions of the reduction reaction of AgNO_3_ (20 cc, 0.02 M), with different quantities of NaBH_4_ (0.02 M): (**d**) 5 cc; (**e**) 10 cc; (**f**) 15 cc; (**g**) 20 cc.

The process of reduction of Ag^+^ to Ag^0^ was followed by UV-Vis spectroscopy (Figure 6). Before starting the reduction reaction, the signals of the reagents were identified. The UV band between 200 and 260 nm was assigned to the AgNO_3_ solution, while the NaBH_4_ solution did not present a signal. By adding different amounts of NaBH_4_ to the AgNO_3_ solution, the formation of a band at about 400 nm was observed. This band was assigned to the surface plasmon resonance (SPR) effect of Ag-NPs [41,42], with a particle size between 20 and 30 nm [43,44], which confirms a correct approximation in the crystallite size calculation discussed in the XRD and TEM section.

**Figure 7:**
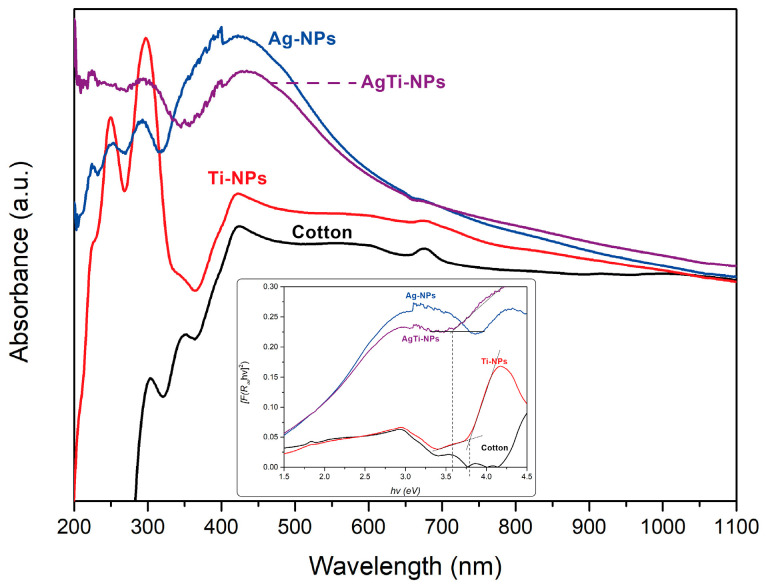
DRS spectra of Ti-NPs, Ag-NPs, and AgTi-NPs samples. Embedded image of Tauc graph for calculating the band gap (dashed lines represent the band gap values).

Figure 7 shows the UV-Vis spectra of the samples with different NPs, obtained using diffuse reflectance spectroscopy (DRS). The band at 410 nm of the Ag-NPs sample confirms the correct synthesis of Ag-NPs on the surface of the cotton fibers, which is directly related to the color change in the fabric from white to brown-golden. On the other hand, the Ti-NPs sample shows bands in the UV region (200–320 nm) attributed to the absorption profile of TiO_2_. For the AgTi-NPs nanocomposite, the characteristic absorption bands of each constituent material were present. However, the TiO_2_ band in the nanocomposite is diminished in intensity and slightly modified in its absorption edge as an effect of the interface with the Ag-NPs.

The calculation of the band gap energy for the samples was performed using the Kubelka–Munk (K–M) function against a Tauc plot (image inserted in Figure 7). The bandgap obtained for the Ti-NPs sample was 3.8 eV, which is a higher value than one of the anatase nanopowders (3.2 eV). It has been reported [45,46] that the band gap values in semiconductors are dependent on the particle size, i.e., when the particle size decreases, the band gap value increases. This phenomenon was probed experimentally by comparing the calculated bandgap of 3.2 eV for the 21 nm TiO_2_ nanopowder (ID 329763580) with the 3.8 eV of the Ti-NPs sample with a particle size of 15 nm, calculated using XRD. Additionally, the AgTi-NPs sample showed a bandgap value for TiO_2_ of 3.55 eV, because the Ag-NPs modify the absorption edge of TiO_2_ due to the interface formed between these materials in the nanocomposite structure. Otherwise, the absorption edge in the visible region of Ag-NPs (bandgap of 2.1 eV) maintains the same behavior for both AgTi-NPs and Ag-NPs samples. Nevertheless, the participation of the energy edge of Ag-NPs in the nanocomposite is to promote electrons excited by low energies (within the Vis spectrum) towards the conduction band of TiO_2_. The intention is to minimize the recombination of the electron/hole (e^−^/h^+^) pair in the photocatalyst and to enhance the photocatalytic activity.

#### Evaluation of the Recombination of the Electron–Hole Pair (e^−^/h^+^) by Photoluminescence (PL)

**Figure 8:**
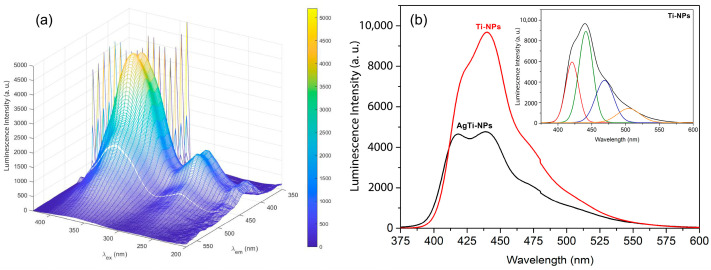
(**a**) Distribution of EEM photoluminescence intensity of the AgTi-NPs. λ_ex_ = excitation wavelength. λ_em_ = emission wavelength. (**b**) Photoluminescence spectrum of Ti-NPs and AgTi-NPs with a λ_ex_ = 350 nm. The image embedded in (**b**) shows the deconvolution of the spectrum of Ti-NPs. The peaked signals behind the 3D plot correspond to the Rayleigh scattering.

The photogenerated charges (e^−^/h^+^) from the absorption of light (photons) with an energy greater than or equal to the band gap of the NPs of Ag and TiO_2_ can recombine and not participate in the interfacial electron transfer (IFET) process, releasing energy in the form of photoluminescence [47]. The Excitation Emission Matrix Technique (EEMF) is a collection of emission and excitation spectra collected at different excitation (λ_ex_) and emission (λ_em_) wavelengths. Figure 8a shows a 3D EMMF plot of the AgTi-NPs sample, where the highest luminescence intensity was obtained at λ_ex_ = 350 nm and λ_em_ = 420 nm. The peaked signals behind the 3D plot correspond to the Rayleigh scattering.

To evaluate the recombination of the e^−^/h^+^ pair in the samples at λ_ex_ = 350 nm, the photoluminescence (PL) was tested for all samples. Results for the Ti-NPs and AgTi-NPs samples are shown in Figure 8b. Hence, a lower PL intensity indicates less charge recombination. The Ti-NPs sample exhibited higher PL intensity, indicating that the recombination rate of the e^−^/h^+^ pair is higher compared to the AgTi-NPs sample. The PL spectrum (inset in Figure 8b) of the Ti-NPs sample is composed of different contributions [48]. The red band (420 nm) corresponds to the indirect recombination of the e^−^/h^+^ pair between the CB and VB through the forbidden band, while the green band (441 nm) is attributed to excitons resulting from vacancies and oxygen defects on the surface (bands at 469 and 504 nm). The presence of Ag-NPs in the nanocomposite greatly reduces exciton recombination in the TiO_2_-NPs as well as indirect recombination. This allows the photogenerated charges on the AgTi-NPs sample to be more available to participate in the IFET reactions necessary for photocatalysis. According to Zhang et al. [49], the Ag-NPs exhibit PL between 330 and 550 nm. Although the Ag-NPs cannot be easily identified in the PL spectrum of the AgTi-NPs sample, their presence decreases the intensity of the bands that make up the PL spectrum of the Ti-NPs sample. Thus, the nanocomposite allows the photogenerated charges to efficiently participate in the interfacial electron transfer reactions of photocatalysis.

### 3.6. Photocatalytic Activity

**Figure 9:**
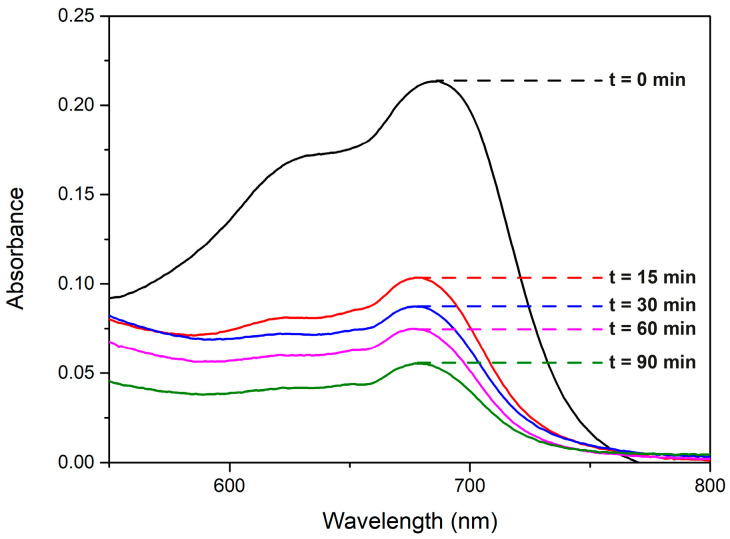
UV-Vis spectra of MB under UV irradiation for the AgTi-NPs samples.

The photocatalytic activities of Ti-NPs, Ag-NPs, and AgTi-NPs samples were evaluated using the MB degradation, using constant and continuous UV irradiation (Hg lamp) or sunlight, as described by Alvarez-Amparán et al. [24]. Previously, squares of fabric of 2.5 × 2.5 cm were impregnated with a solution of 5 ppm of MB. The MB shows a maximum absorption band in the visible region at 664 nm. The photocatalytic reaction was followed by UV-Vis (DRS) spectra taken at intervals of 0, 15, 30, 60, and 90 min. The degradation of MB on the AgTi-NPs samples was determined by the C/C_0_ ratio, as mentioned above. Figure 9 shows the change in the UV-Vis spectrum (DRS) of MB as a function of reaction time for AgTi-NPs under UV irradiation. The degradation of MB obtained after 90 min was 75%, which is a higher value than that obtained for the cotton fabric alone, where the degradation of MB was only 21% at 90 min. Thus, AgTi-NPs easily triple the MB degradation, showing that the nanocomposite possesses an important photocatalytic activity under UV irradiation.

**Figure 10:**
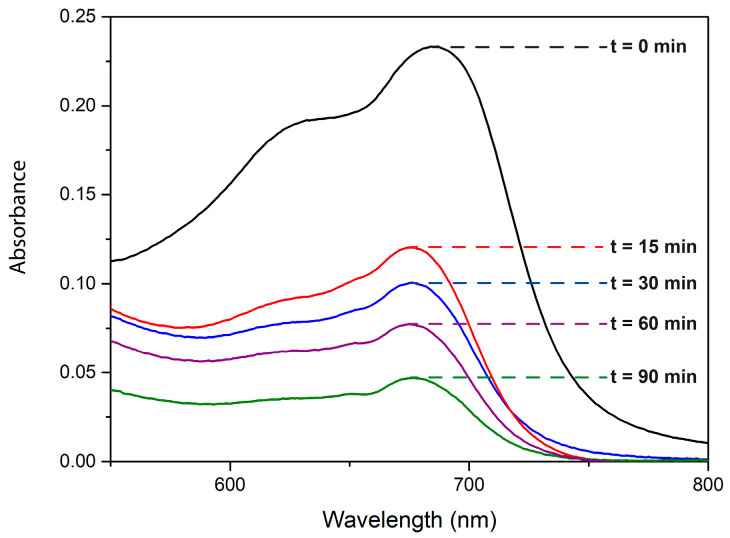
UV-Vis spectra of MB under solar irradiation for AgTi-NPs samples.

MB degradation was also evaluated for the AgTi- NPs samples using solar irradiation (Figure 10), and 80% of degradation was achieved after 90 min. At the same reaction time, MB degradation in the cotton fibers was only 36%. This confirms an important photocatalytic activity of the nanocomposite for MB degradation under sunlight.

**Scheme 2:**
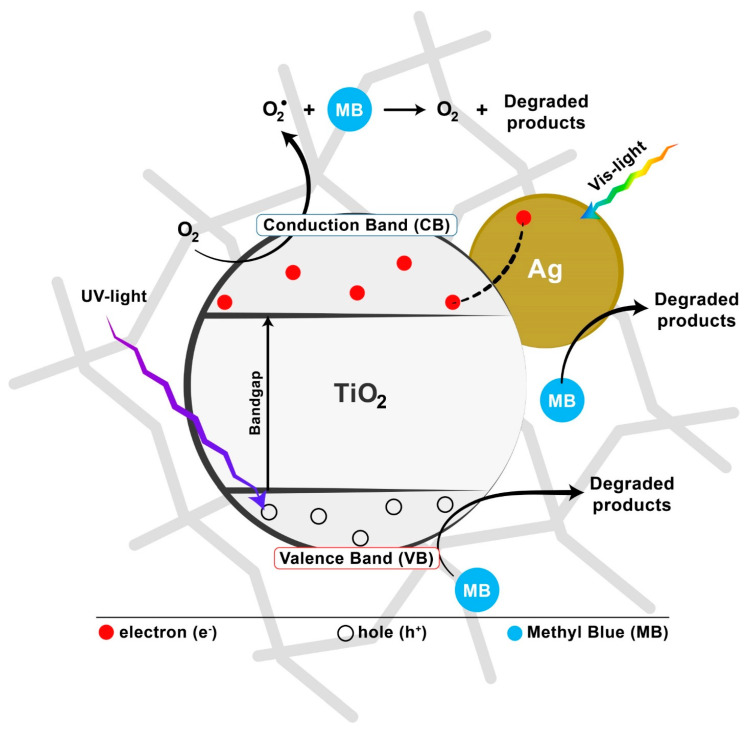
MB degradation pathways using AgTi-NPs with UV- and Vis-light.

It is important to note that the power density for each photocatalytic reaction was different. The power density was 10 mW/cm^2^ for the UV radiation and 3 mW/cm^2^ for the solar irradiation, according to the climatic conditions of the experimental day in Mexico City. This means that the Ag-NPs in the nanocomposite promote low-energy excited electrons formed by absorbing photons from the visible spectrum to migrate towards the conduction band (BC) of TiO_2_. This allows the nanocomposite to compete and achieve MB degradation values very close to those obtained by UV irradiation, using only sunlight. Scheme 2 presents the possible photodegradation pathways of MB using AgTi-NPs as photocatalysts.

**Figure 11:**
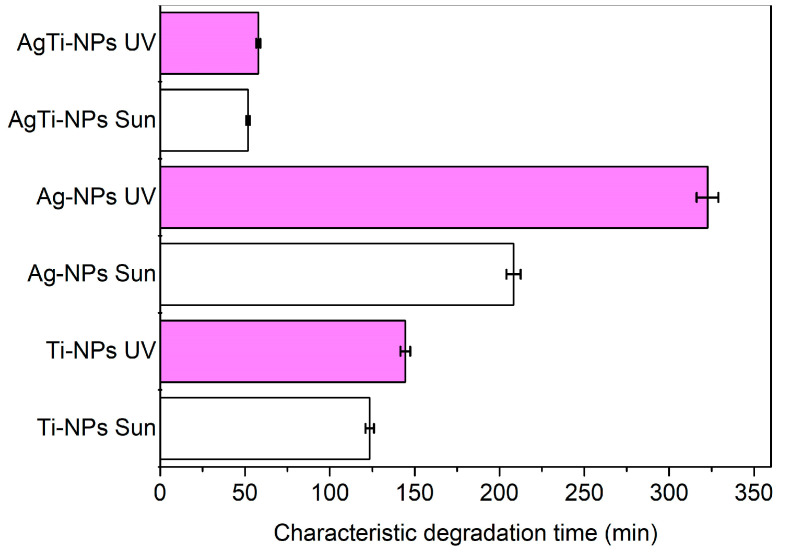
Characteristic degradation time (τ) of MB, under UV (magenta) or solar (sun) irradiation (white).

The kinetic data of MB degradation were fitted to a first-order pseudokinetic model. The Ag-NPs sample showed kinetic coefficients of 0.0031 and 0.0048 min^−1^ under UV and solar irradiation, respectively. Meanwhile, for the AgTi-NPs samples the obtained kinetic coefficients were higher with values of 0.0173 and 0.0193 min^−1^ for UV and solar irradiation, respectively. These values indicate that the Ag-NPs do not possess significant photocatalytic activity alone for MB degradation. This may be attributed to the high recombination of photogenerated electrons in Ag-NPs. On the other hand, the increase in the kinetic coefficient of Ag-NPs using solar versus UV irradiation is attributed to an additional amount of photogenerated electrons coming from the low energies of the visible spectrum. The photocatalytic activity of the Ti-NPs sample in MB degradation under solar irradiation was also evaluated, and the value of the kinetic coefficient was 0.0081 min^−1^ compared with the results of UV irradiation (obtained in previous work [24] and shown in Figure 11), where the Ti-NPs sample also presents less photoactivity. These photo activities were 2.5 and 2 times lower than those obtained with the AgTi-NPs sample under UV and solar irradiation, respectively. This shows that the nanocomposite has a lower recombination of the e^−^/h^+^ pair compared to the individual materials of which it is composed. This allows a higher interfacial electron transfer (IFET) with the MB molecules for their photocatalytic degradation.

Figure 11 shows that for all the samples the characteristic MB degradation time (τ) was lower under solar irradiation than under UV irradiation.

### 3.7. Antibacterial Properties of Fabrics

A sunlight exposure assay was performed where the textiles were exposed to a radiation of 97 Klux and a UV AB radiation of 7525 µW/cm^3^ under the glass microscope slide.

**Figure 12:**
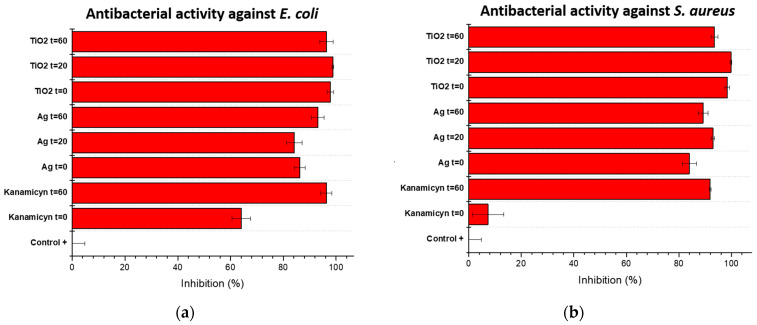
Antibacterial activity of cotton fabrics against (**a**) Gram − (*E. coli*) and (**b**) Gram + (*S. aureus*) bacteria with and without sunlight radiation in solution after 18 h of incubation. Symbol t shows the time of sunlight exposure in minutes.

Both Ag/cotton and Ag–TiO_2_ composites showed good antibacterial activity against both *E. coli* and *S. aureus* with inhibition higher than 80%, and up to 95%. Both types of cotton nanocomposites showed better inhibition properties than the kanamycin reference sample. In solution, both bacteria were susceptible to the nanocomposites on cotton fabrics as shown in Figure 12. *S. aureus* (Figure 12b) showed a slightly higher susceptibility to both composite treatments. However, the Ag–TiO_2_/cotton composite presented better overall antibacterial activity against both strains of bacteria. Interestingly, kanamycin showed better antibacterial activity after 60 min of sunlight exposure compared to the same antibiotic treatment without sunlight exposure. This effect might be caused by an increase in the interaction of the antibiotic with the bacteria during the period of sunlight exposure before submerging the fabric into a sterile LB medium where the antibiotic–bacteria interaction may be reduced due to the liquid medium since the kanamycin concentration is possibly reduced considerably by the liquid medium.

On the other hand, Ag/cotton and Ag–TiO_2_/cotton composites retain a similar biocidal effect at differing times of sunlight exposure. It is possible that, at this bacterial concentration and exposure time, the composites’ effect cannot be differentiated between the varying periods of sunlight irradiation.

As previously reported, the antibacterial activity of fabrics impregnated with nanoparticles cannot be evaluated using the traditional Kirby–Bauer method because the lack of nanoparticle diffusion on LB agar does not allow for the formation of inhibition zones [24]. Hence, a reverse assay was proposed where the fabrics with nanoparticles were directly inoculated with bacterial cultures and then deposited on LB agar plates to analyze bacterial growth. Using the previously described procedure, we qualitatively analyzed the antibacterial properties of the Ag/cotton and Ag–TiO_2_/cotton composites. Generally, both bacteria were seen to be susceptible to nanoparticle treatment with sunlight exposure. At t = 0/without solar irradiation, the fabrics containing antibiotics and nanoparticles showed bacterial growth. However, *S. aureus* growth was inhibited in fabrics containing Ag–TiO_2_. This phenomenon could be explained by the additive effect of the combination of Ag and TiO_2_ nanoparticles. Additionally, Gram-positive bacteria such as *S. aureus* lack the characteristic outer membrane present in Gram-negative bacteria [47]. The absence of this membrane makes Gram-positive bacteria more susceptible to many antibiotics and, possibly, to antibacterial nanoparticles.

**Figure 13:**
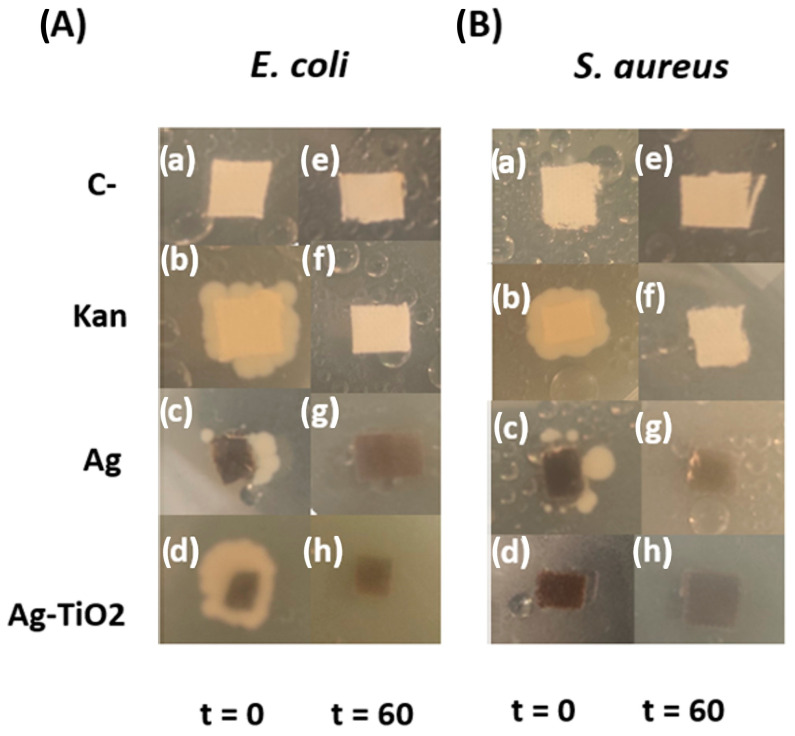
Fabrics inoculated with *E. coli* (**A**) and *S. aureus* (**B**) cultures with and without solar irradiation on LB agar plates after 40 h of incubation. Symbol t shows the time of sunlight exposure in min.

At 20 h of incubation in LB agar plates, the fabrics inoculated with bacteria with and without solar irradiation show similar growth to the growth seen in liquid culture. After 60 h of incubation, the bacteria begin to grow more rapidly and the previously seen antibacterial effect diminishes, as shown in Figure 13A(b–d),B(b–d). It could be possible that the surviving bacteria begin spreading on the agar and gain access to more nutrients while also moving away from the biocides present on the textiles. This results in a diminishing interaction between the bacteria and biocides, hence allowing for the bacteria to grow more rapidly. This hypothesis is supported by the results obtained using liquid cultures (Figure 12), where *E. coli* exposed to biocides shows a growth percentage of 4% for kanamycin, 14% for Ag/cotton composites, and 3% for Ag–TiO_2_/composites. Meanwhile, liquid cultures of *S. aureus* show a growth percentage of 8% for kanamycin, 10% for Ag/cotton composites, and 6% for Ag–TiO_2_/cotton composites.

## 4. Conclusions

In this work, we report an effective method to functionalize cotton fabrics in situ with TiO_2_ and Ag nanocomposites resulting in a good dispersion of nanoparticles on the fabric. A cooperative effect was found in the photoluminescence experiment showing a decreased signal in the presence of Ag–TiO_2_ with respect to TiO_2_ alone. This is because Ag-NPs modify the absorption edge of TiO_2_, due to the interface formed between these materials in the nanocomposite structure.

The photocatalytic effect on the degradation of methylene blue was improved by the presence of both types of nanoparticles, Ag and TiO_2_, in the presence of UV as well as sunlight radiation. The rate of MB degradation in the Ag–TiO_2_ composites was three times higher than in TiO_2_ alone, showing a synergic effect between Ag and TiO_2_. This photocatalytic effect agrees with the decrease in the band gap and with the diminished signal of photoluminescence. In conclusion, a synergic effect was observed for both types of irradiation, UV and visible light, and the degradation rate of MB was higher for visible light than for UV.

Thus, the functionalization of fabrics with Ag and TiO_2_ nanoparticles has potential interest to improve the efficiency of photocatalysts under a wide range of wavelengths. Furthermore, these results indicate the potential applications of functionalized fabrics prepared using ultrasound-assisted and solvothermal successive methods to deposit nanoparticles onto them, where these fabrics can be used as more efficient photocatalytic materials.

On the other hand, the fabrics impregnated with Ag and Ag–TiO_2_ nanoparticles also showed an excellent biocide effect against model Gram-negative and Gram-positive bacteria showing inhibition rates higher than 80%. Therefore, this technique could be optimal for the fabrication of personal protection equipment (PPE) due to the improved antibacterial properties of fabric composites obtained.

## Data Availability

Not applicable.

## References

[1] Hussain C.M. (2020). Handbook of Nanomaterials for Manufacturing Applications.

[2] Patil M.P., Kim G.-D. (2017). Eco-friendly approach for nanoparticles synthesis and mechanism behind antibacterial activity of silver and anticancer activity of gold nanoparticles. Appl. Microbiol. Biotechnol..

[3] Sarkar J., Achary K. (2017). Alternaria alternata culture filtrate mediated bioreduction of chloroplatinate to platinum nanoparticles. Inorganic Nano-Met. Chem..

[4] Kästner C., Thünemann A.F. (2016). Catalytic Reduction of 4-Nitrophenol Using Silver Nanoparticles with Adjustable Activity. Langmuir.

[5] Guo Q., Zhou C., Ma Z., Yang X. (2019). Fundamentals of TiO_2_ Photocatalysis: Concepts, Mechanisms, and Challenges. Adv. Mater..

[6] Ruban A., Hammer B., Stoltze P., Skriver H.L., Nørskov J.K. (1997). Surface Electronic Structure and Reactivity of Transition and Noble Metals. J. Mol. Catal. A Chem..

[7] Yocupicio-Gaxiola R.I., Petranovskii V., Sánchez P., Antúnez-García J., Alonso-Núñez G., Galván D.H., Murrieta-Rico F.N. (2021). Prospects for Further Development of Face Masks to Minimize Pandemics—Functionalization of Textile Materials with Biocide Inorganic Nanoparticles: A Review. IEEE Lat. Am. Trans..

[8] Senić Ž., Bauk S., Vitorović-Todorović M., Pajić N., Samolov A., Rajić D. (2011). Application of TiO_2_ Nanoparticles for Obtaining Self Decontaminating Smart Textiles. Sci. Tech. Rev..

[9] Ogunsona E.O., Muthuraj R., Ojogbo E., Valerio O., Mekonnena T.H. (2020). Engineered nanomaterials for antimicrobial applications: A review. Appl. Mater. Today.

[10] Kim J.S., Kuk E., Yu K.N., Kim J.H., Park S.J., Lee H.J., Kim S.H., Park Y.K., Park Y.H., Hwang C.Y. (2007). Antimicrobial effects of silver nanoparticles. Nanomedicine.

[11] Yin I.X., Zhang J., Zhao S.I., Mei M.L., Li Q., Chu C.H. (2020). The antibacterial mechanism of silver nanoparticles and its application in dentistry. Int. J. Nanomed..

[12] Maiti S., Krishnan D., Barman G., Ghosh S.K., Laha J.K. (2014). Antimicrobial activities of silver nanoparticles synthesized from Lycopersicon esculentum extract. J. Anal. Sci. Technol..

[13] Pietrzak K., Gutarowska B., Machnowski W., Mikołajczyk U. (2016). Antimicrobial properties of silver nanoparticles misting on cotton fabrics. Text. Res. J..

[14] Kalwar K., Shan D. (2018). Antimicrobial effect of silver nanoparticles (AgNPs) and their mechanism–a mini review. Micro Nano Lett..

[15] Bruna T., Maldonado-Bravo F., Jara P., Caro N. (2021). Silver Nanoparticles and Their Antibacterial Applications. Int. J. Mol. Sci..

[16] Hiragond C.B., Kshirsagar A.S., Dhapte V.V., Khanna T., Joshi P., More P.V. (2018). Enhanced anti-microbial response of commercial face mask using colloidal silver nanoparticles. Vacuum.

[17] Kharaghani D.M., Khan A., Shahzad Y., Inoue T., Yamamoto S., Rozet Y., Tamada Y., Kim I.S. (2018). Preparation and In-Vitro Assessment of Hierarchal Organized Antibacterial Breath Mask Based on Polyacrylonitrile/Silver (PAN/AgNPs) Nanofiber. Nanomaterials.

[18] Guo W., Lin Z., Wang X.G. (2003). Sonochemical synthesis of nanocrystalline TiO_2_ by hydrolysis of titanium alkoxides. Microelectron. Eng..

[19] Prasad K., Pinjari D.V., Pandit A.B., Mhaske S.T. (2010). Synthesis of titanium dioxide by ultrasound assisted sol–gel technique: Effect of amplitude (power density) variation. Ultrason. Sonochem..

[20] Hadad L., Perkas N., Gofer Y., Calderon-Moreno J., Ghule A., Gedanken A. (2007). Sonochemical deposition of silver nanoparticles on wool fibers. J. Appl. Polym. Sci..

[21] Perelshtein I., Applerot G., Perkas N., Wehrschuetz-Sigl E., Hasmann A., Guebitz G., Gedanken A. (2009). Antibacterial Properties of an In Situ Generated and Simultaneously Deposited Nanocrystalline ZnO on Fabrics. Appl. Mater. Inter..

[22] Perelshtein I., Applerot G., Perkas N., Wehrschuetz-Sigl E., Hasmann A., Guebitz G., Gedanken A. (2009). CuO–cotton nanocomposite: Formation, morphology, and antibacterial activity. Surf. Coat. Technol..

[23] Perelshtein I., Applerot G., Perkas N., Grinblat J., Gedanken A. (2012). A One-Step Process for the Antimicrobial Finishing of Textiles with Crystalline TiO_2_ Nanoparticles. Chem. Eur. J..

[24] Alvarez-Amparán M.A., Martínez-Cornejo V., Cedeño-Caero L., Hernandez-Hernandez K.A., Cadena-Nava R.D., Alonso-Núñez G., Fuentes Moyado S. (2022). Characterization and photocatalytic activity of TiO_2_ nanoparticles on cotton fabrics, for antibacterial masks. Appl. Nanosci..

[25] Akhavan F., Montazerb S.M. (2012). In situ sonosynthesis of nano TiO_2_ on cotton fabric. Ultrason. Sonochem..

[26] Abulikemu M., Tabrizi B., Ghobadioo S., Mofarah H., Jabbour G. (2021). Silver Nanoparticle-Decorated Personal Protective Equipment for Inhibiting Human Coronavirus Infectivity. ACS Appl. Nano Mater..

[27] Dong H., Hinestroza J.P. (2009). Metal Nanoparticles on Natural Cellulose Fibers: Electrostatic Assembly and In Situ Synthesis. ACS Appl. Mater. Interfaces.

[28] Pakdel E., Daoud W.A. (2013). Self-cleaning cotton functionalized with TiO_2_/SiO_2_: Focus on the role of silica. J. Colloid. Interface.

[29] Chung C., Lee M., Choe E.K. (2004). Characterization of cotton fabric scouring by FT-IR ATR spectroscopy. Carbohydr. Polym..

[30] Abidi N., Cabrales L., Haigler C.H. (2014). Changes in the cell wall and cellulose content of developing cotton fibers investigated by FTIR spectroscopy. Carbohydr. Polym..

[31] Âkerholm M., Hinterstoisser B., Almén L. (2004). Characterization of the crystalline structure of cellulose using static and dynamic FT-IR spectroscopy. Carbohydr. Res..

[32] Md Salim R., Asik J., Sarjadi M.S. (2021). Chemical functional groups of extractives, cellulose and lignin extracted from native Leucaena leucocephala bark. Wood Sci. Technol..

[33] El-Sakhawy M., Kamel S., Salama A., Tohamy H.A.S. (2018). Preparation and infrared study of cellulose based amphiphilic materials. Cellul. Chem. Technol..

[34] Ivanova T., Harizanova A., Koutzarova T., Vertruyen D.B. (2016). Characterization of nanostructured TiO_2_: Ag films: Structural and optical properties. J. Phys. Conf. Ser..

[35] Andrés E.S., Toledano-Luque M., Prado A.D., Navacerrada M.A., Mártil I., González-Díaz G., Strub E. (2005). Physical properties of high pressure reactively sputtered TiO_2_. J. Vac. Sci. Technol. A Vac. Surf. Film..

[36] French A.D. (2014). Idealized powder diffraction patterns for cellulose polymorphs. Cellulose.

[37] Martínez Espinosa J.C., Carrera Cerritos R., Ramírez Morales M.A., Sánchez Guerrero K.P., Silva Contreras R.A., Macías J.H. (2020). Characterization of silver nanoparticles obtained by a green route and their evaluation in the bacterium of Pseudomonas aeruginosa. Crystals.

[38] Lopes F.C.S., Maria da Graça C., Bargiela P., Ferreira H.S., Pires C.A.D.M. (2020). Ag/TiO_2_ photocatalyst immobilized onto modified natural fibers for photodegradation of anthracene. Chem. Eng. Sci..

[39] Wang G., Shi C., Zhao N., Du X. (2007). Synthesis and characterization of Ag nanoparticles assembled in ordered array pores of porous anodic alumina by chemical deposition. Mater. Lett..

[40] Zahornyi M.M., Tyschenko N.I., Lobunets T.F., Kolomys O.F., Strelchuk V.V., Naumenko K.S., Biliavska L.O., Zahorodnia S.D., Lavrynenko O.M., Ievtushenko A.I. (2021). The Ag influence on the surface states of TiO_2_, optical activity and its cytotoxicity. J. Nano-Electron. Phys..

[41] Jensen T.R., Malinsky M.D., Haynes C.L., Van Duyne R.P. (2000). Nanosphere lithography: Tunable localized surface plasmon resonance spectra of silver nanoparticles. J. Phys. Chem. B.

[42] Mock J.J., Barbic M., Smith D.R., Schultz D.A., Schultz S. (2002). Shape effects in plasmon resonance of individual colloidal silver nanoparticles. J. Chem. Phys..

[43] Agnihotri S., Mukherji S., Mukherji S. (2014). Size-controlled silver nanoparticles synthesized over the range 5–100 nm using the same protocol and their antibacterial efficacy. Rsc Adv..

[44] Paramelle D., Sadovoy A., Gorelik S., Free P., Hobley J., Fernig D.G. (2014). A rapid method to estimate the concentration of citrate capped silver nanoparticles from UV-visible light spectra. Analyst.

[45] Lin H., Huang C.P., Li W., Ni C., Shah S.I., Tseng Y.H. (2006). Size dependency of nanocrystalline TiO_2_ on its optical property and photocatalytic reactivity exemplified by 2-chlorophenol. Appl. Catal. B Environ..

[46] Kumar S., Verma N.K., Singla M.L. (2012). Size dependent reflective properties of TiO_2_ nanoparticles and reflectors made thereof. Dig. J. Nanomater. Biostruct..

[47] Breijyeh Z., Jubeh B., Karaman R. (2020). Resistance of Gram-Negative Bacteria to Current Antibacterial Agents and Approaches to Resolve It. Molecules.

[48] Saha A., Moya A., Kahnt A., Iglesias D., Marchesan S., Wannemacher R., Prato M., Vilaveta J.J., Guldi D.K. (2017). Interfacial charge transfer in functionalized multi-walled carbon nanotube@TiO_2_ nanofibres. Nanoscales.

[49] Zhang A., Zhang J., Fang Y. (2008). Photoluminescence from colloidal silver nanoparticles. J. Lumin..

